# How do ophthalmologists manage functional visual symptoms? A UK survey of ophthalmologists’ experience

**DOI:** 10.1007/s00417-021-05433-4

**Published:** 2021-10-11

**Authors:** Masara Laginaf, Harry Costello, Gary Price

**Affiliations:** 1 Luton &, Dunstable University Hospital, Luton, UK; 2 Institute of Cognitive Neuroscience, University College London (UCL), London, UK; 3 The National Hospital for Neurology and Neurosurgery, London, UK

**Keywords:** Functional visual loss, Nonorganic visual loss

## Abstract

**Background/aims:**

Functional visual symptoms are relatively common symptoms seen by ophthalmologists. However, there are no consensus guidelines on ophthalmological management of this condition, and there is a paucity of knowledge about the collective challenges experienced in treating patients with functional visual symptoms. In order to establish an ophthalmological perspective on this condition, we undertook the first national survey of experience, knowledge and management of functional visual symptoms amongst ophthalmologists.

**Methods:**

An online survey was disseminated to ophthalmologists in the UK via all Royal College of Ophthalmology college tutors.

**Results:**

One hundred nineteen ophthalmologists completed the survey. Functional visual symptoms accounted for 3% of all new referrals. Forty per cent of respondents felt they had a good understanding of functional visual symptoms. Two-thirds reported a need for further training in this area. Respondents estimated two-thirds of patients’ symptoms improved, but a third experienced severe or extreme disability. Following diagnosis, a minority of patients were referred to mental health or neurology services. The majority of respondents described difficulty discussing psychological factors, with a lack of time or space in a clinic preventing a holistic approach. Free text comments highlighted a lack of access to dedicated psychological support for patients.

**Conclusion:**

Functional visual symptoms are disabling and are seen relatively frequently by ophthalmologists. This preliminary survey suggests that care pathways for patients with functional visual symptoms could be optimised. Fostering links between ophthalmology and existing services with expertise in functional disorders could improve patient care and clinician education and ultimately encourage research in this area.

**Supplementary Information:**

The online version contains supplementary material available at 10.1007/s00417-021-05433-4.


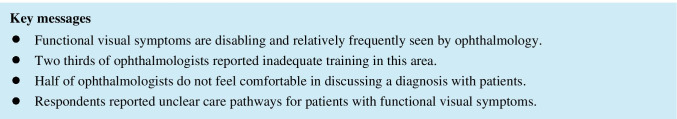


## Introduction

Functional visual symptoms are a recognised presentation of conversion or functional neurological symptom disorder. The Diagnostic and Statistical Manual of Mental Disorders (DSM–5) diagnostic criteria defines functional neurological symptoms as those affecting voluntary motor or sensory function in the absence of disease or incompatibility with underlying disease [1]. Though stressful life events are significant risk factors for developing functional symptoms [2], preceding conflicts or other stressors to the onset of symptoms have been excluded as a necessary criterion for diagnosis in the latest DSM-5. Cases of functional visual loss, also referred to as “nonorganic visual loss”, and previously described as “hysterical blindness”, have been reported in the literature dating back to the eighteenth century [3].

While there has been increasing focus and research on the impact and most effective treatment of functional neurological disorders, including the development of national specialist services, relatively little attention has been paid to functional visual conditions [4, 5]. Functional neurological disorders are estimated to account for 16% of new referrals to neurologists [6], have significant care costs and are equally as disabling and distressing to patients as other neurological conditions including epilepsy and multiple sclerosis [7]. However, few studies to date have established the prevalence and impact of functional visual loss on patients.

It is estimated that functional visual loss accounts for 5% of general ophthalmology clinic cases [8], increasing to 12% of patient diagnoses in neuro-ophthalmology clinics [9]. Diagnosis of functional visual loss can be challenging for clinicians and should be supported by positive findings on examination rather than be a diagnosis of exclusion. This can be complicated due to the high frequency of co-existent organic disease reported in up to half of patients [9, 10].

Though few longitudinal studies measuring outcomes have been conducted, significant disability and enduring symptoms have been reported in up to 60% of patients with functional visual loss [11, 12]. Case series have described individual therapeutic approaches for patients with functional visual loss [13]; however, the efficacy of these interventions remains unclear, and the recovery rate has been reported to be as low as 25% [12].

Functional disorders occur in every medical specialty and now occupy a standard part of curriculum for training, practice and research in several specialties including neurology and gastroenterology [14]. There is increasing understanding of the importance of the initial assessment, explanation and patient education as a crucial therapeutic step in the treatment of these disorders [14]. Significant research has explored the experience and management approaches of neurologists and other healthcare professionals when assessing and treating patients with functional symptoms [15, 16]. Although ophthalmologists regularly assess and are the first to deliver a diagnosis of functional visual loss, no study to date has sought to quantify ophthalmologists’ understanding and management of these patients. This is the first national survey of ophthalmologists that has sought to address this issue.

## Methods

An online survey was designed collaboratively between ophthalmology and neuropsychiatry clinicians. The survey was constructed based on previous surveys of other health professionals regarding functional symptoms [15, 16]. The survey contained 30 questions, mostly multiple choice but with some free text, first covering demographics and details of clinician’s practice and then addressing their understanding, management of and attitudes to functional visual symptoms. The degree of functional impairment was rated based on the scale used in the World Health Organization (WHO) disability assessment schedule [17].

The survey was sent via email to 111 ophthalmologists across the UK who were registered as college tutors with the Royal College of Ophthalmologists (RCOphth). The tutors were asked to complete and disseminate the survey across their respective training deaneries and local trusts. The survey was available online for completion using the secure survey and questionnaire tool Opinio. It was open for completion for a time limited period of 3 months from June to August 2020. All answers were fully anonymised.

## Results

### Respondents and experience (see Table 1)

**Table 1:** Respondents

		***n***	**%**
Clinical grade	ST1-3	20	17.1
	ST4 and above	24	20.5
	Specialty doctor	18	15.4
	Fellow	2	1.7
	Consultant	54	46.2
Subspecialty	General	56	38.4
	Neuro-ophthalmology	12	8.2
	Paediatrics	21	14.4
	Medical retina	16	11.0
	Vitreoretinal	10	6.8
	Glaucoma	16	11.0
	Anterior segment	7	4.8
	Oculoplastics	2	1.4
	Primary care ophthalmology	6	4.1
Years working in ophthalmology	< 5 years	29	24.8
	5–10 years	27	23.1
	10–15 years	14	12.0
	15–20 years	10	8.5
	> 20 years	38	32.5
Location in the UK practicing	England	105	89.7
	Wales	7	6.0
	Scotland	6	5.1
	Northern Ireland	0	0.0
Completed medical training in the UK	Yes	82	71.3
	No	34	29.6

One hundred and nineteen ophthalmologists completed the survey. Almost half of respondents were consultants (46.2%), and ophthalmologists in training accounted for a third of responses. There was a wide range in the number of years’ experience in ophthalmology, with around a third of respondents reporting over 20 years’ experience and half of respondents reporting 10 years or less. The majority (38.4%) of respondents were general ophthalmologists, followed by paediatric ophthalmologists (14.4%). General ophthalmology was the most common respondent subspecialty, and this was probably due to trainee respondents, who were yet to sub-specialise. Neuro-ophthalmology accounted for only 8.2% of respondents. The vast majority of respondents (89.7%) practised in England and had completed medical training in the UK (71.3%).

### Presentation, management and outcomes of functional visual symptoms (see Table 2)

**Table 2:** Presentation, management and outcomes of functional visual symptoms

	*n*	Mean (SD)	Median (IQR)
What % of new referrals to clinic has functional visual symptoms?	114	2.9 (2.9)	2 (1–5)
What % of patients with functional visual symptoms has bilateral symptoms?	112	43.2 (31.7)	50 (10–70)
How many appointments will a patient with functional visual symptoms typically have?	116	3.9 (1.8)	3 (3–5)
What % of ADULTS with functional visual symptoms will you follow up after diagnosis?	114	31.0 (37.0)	10 (0–50)
What % of CHILDREN with functional visual symptoms will you follow up after diagnosis?	112	52.7 (42.4)	50 (1–100)
What % of patients with functional visual symptoms gets better?	108	61.9 (30.5)	70 (50–90)
What is the most common presentation of functional visual symptom?	n	%	
Reduced vision	79	67.5	
Visual field loss	5	4.3	
Combined reduced vision and visual field loss	31	26.5	
Visual snow or “static”	1	0.9	
Diplopia	1	0.9	
Photophobia	1	0.9	
How impaired by their symptoms are patients with functional visual symptoms?			
No impairment	5	4.3	
Mild impairment	30	25.6	
Moderate impairment	49	41.9	
Severe impairment	29	24.8	
Extreme impairment	5	4.3	
What investigations do you usually perform for patients with functional visual symptoms?			
Humphrey visual field test	89	22.6	
Goldmann visual field test	40	10.2	
Neuroimaging	75	19.1	
Electrodiagnostics	68	17.3	
Optical coherence tomography	97	24.7	
Fundus fluorescein angiogram	9	2.3	
Other	19	4.8	
Do patients you see with functional visual symptoms usually have other functional symptoms?			
Yes	30	25.9	
No	49	42.2	
Do not know	38	32.8	
What management plan do you typically make for patients with functional visual symptoms?			
Discharge to GP	33	28.2	
Follow-up in clinic	51	43.6	
Refer to psychological or psychiatric services	20	17.1	
Refer to neurology	14	12.0	
Interquartile range (IQR), standard deviation (SD), general practitioner (GP), percentage (%)			

The mean estimated percentage of referrals seen in clinic with functional visual symptoms was 2.9%, which is lower than the prevalence reported in other studies of around 5%. The survey revealed that symptoms are bilateral in half of cases, with the most common presentation being reduced vision (67.5%). Respondents reported the majority of patients (41.9%) experienced moderate impairment in function due to their symptoms, but almost a third (29.1%) suffered severe or extreme impairment.

The most common investigation used by respondents in patients with functional visual symptoms was optical coherence tomography (24.7%), though a third of respondents used some form of visual field testing (Humphrey 22.6%, Goldmann 10.2%) and almost a fifth of patients underwent further neuroimaging.

The majority of respondents (42.2%) reported functional visual symptoms presenting as an isolated functional symptom, though a third of respondents did not know whether other functional symptoms were also present.

On average, patients with functional visual symptoms had three appointments with respondents prior to discharge. Children (52.7%) were more likely to be followed up after diagnosis than adults (31%).

Almost two-thirds (61.9%) of patients’ symptoms were estimated to improve. The most common reported management plan was monitoring and follow-up in clinic (43.6%). A third of patients were discharged to their general practitioner (GP). A fifth of respondents were typically referred on to mental health services and even fewer referred for neurology input (14%).

### Attitudes, knowledge and beliefs regarding functional visual symptoms (see Table 3)

**Table 3:** Attitudes, knowledge and beliefs regarding functional visual symptoms

		Strongly disagree	Disagree	Neither agree nor disagree	Agree	Strongly agree
I have a good knowledge of functional visual disorders	*n*	7	20	44	37	10
	%	6.0	17.1	37.6	31.6	8.5
I received adequate education about functional visual symptoms as part of my training	*n*	7	34	38	34	4
	%	6.0	29.3	32.8	29.3	3.4
Generally I am confident diagnosing functional visual disorders	*n*	6	20	29	55	8
	%	5.1	17.1	24.8	47.0	6.8
These patients’ symptoms are real	*n*	2	10	34	57	13
	%	1.7	8.7	29.6	49.6	11.3
It is appropriate for me to be involved in the diagnosis of functional visual symptoms	*n*	0	6	21	72	17
	%	0.0	5.2	18.3	62.6	14.8
It is appropriate for me to be involved in the treatment of patients with functional visual symptoms	*n*	3	26	31	50	8
	%	2.6	22.2	26.5	42.7	6.8
If I had a choice I would rather not see patients with functional visual symptoms	*n*	13	25	31	37	12
	%	11.1	21.4	26.5	31.6	10.3
Generally I am comfortable explaining the diagnosis of a functional disorder to a patient	*n*	5	25	22	57	9
	%	4.3	21.4	18.8	48.7	7.7
I often struggle with the discussion of associated psychiatric/psychological problems	*n*	4	31	29	47	7
	%	3.4	26.5	24.8	40.2	6.0
I am confident discussing the possibility of a functional visual disorder with a patient	*n*	1	28	29	49	10
	%	0.9	24.1	25.0	42.2	8.6
Patients with functional visual impairment should be allowed to drive	*n*	17	56	40	5	0
	%	14.5	47.9	34.2	4.3	0.0
Disability benefits should not be awarded to these patients because it will prevent them from getting better	*n*	2	24	54	32	5
	%	1.7	20.7	46.6	27.6	4.3
Patients with functional visual loss can be registered as sight impaired	*n*	12	38	46	20	2
	%	10.3	32.5	39.3	17.1	1.7

Sixty per cent of ophthalmologists were unsure or disagreed that they had a good knowledge of functional visual symptoms. Only a third of respondents felt they had adequate teaching on functional visual symptoms during their training.

Respondents were fairly evenly split between those who felt confident in diagnosing functional visual disorders (53.8%) and those who were unsure or did not feel confident (46.2%). However, the majority agreed or strongly agreed that it was appropriate for ophthalmologists to be involved in diagnosis (77.4%). Half of all participants agreed that it was appropriate for ophthalmologists to be involved in treating functional visual symptoms, and half thought it was inappropriate to be involved.

Half (50.8%) of respondents agreed or strongly agreed that they were confident in discussing a diagnosis of functional visual disorder with a patient. A quarter were unsure when asked if they struggled to discuss or explore possible psychological stressors contributing to symptoms. This finding may explain why only around a third (32.5%) of respondents would continue to see patients with functional visual symptoms if given a choice.

The majority of participants (60.9%) agreed or strongly agreed that patients’ symptoms were “real”. A third of participants agreed or strongly agreed disability benefits should not be awarded to patients with functional visual symptoms due to concerns it will prevent recovery.

Most respondents (62.4%) either disagreed or strongly disagreed that patients with functional visual impairment should be allowed to drive. However, when asked if patients with functional visual loss could be registered as sight impaired, responses were mixed with the majority split between disagreeing (42.8%) and unsure (39.3%), and only a fifth agreed that they could be registered sight impaired.

### Free text comments

Forty (33.6%) respondents wrote comments with several themes prominent. A number expressed dissatisfaction with access to psychological support and insufficient clinic time or appropriate space to explore factors that may have contributed to symptoms: *“We* have almost no access to psychological services and limited clinic capacity so management is very difficult”, “better pathways for management of these patients are needed”, “I find discussions and management of these patients difficult and unsatisfactory and I do not feel they are adequately managed due to lack of resources for this group”, “…this is extremely difficult as there is not sufficient psychology support for patients”, “…time doesn’t permit proper addressing of all related issues, and can easily take an hour in clinic”.

Comments also highlighted further training needs: “Trainees need to be taught how to approach the management, investigation and diagnosis of potential functional visual symptoms in patients”, “…it would be helpful to have further training in this area and a clearer pathway when this is suspected”, “functional visual loss isn’t covered in detail as part of training”.

Other comments highlighted the differences in outcomes and management between adults and children: “Children tend to get better quicker”, “management is different between adult and child”.

Respondents also noted the vital issue of the overlay of functional symptoms on a background of organic pathology: “I said functional visual symptoms account for 3% of my new referrals, but if I counted my patients with functional overlay it would be much higher”, “half have some form of pathology so often it’s about teasing out which is which and patient concerns”.

## Discussion

Ophthalmologists are at the forefront in the diagnosis and management of patients with functional visual symptoms. This is the first national survey of ophthalmologists’ understanding, attitudes and experience of managing this group of patients.

We found that ophthalmologists see patients with functional visual symptoms relatively frequently and this condition has significant demands on care in terms of patient follow-up. Symptoms are often isolated, typically presenting as reduced vision and resulting in significant disability. Though most patients are followed up by ophthalmology following diagnosis, some are not, and their outcome is unknown. Treatments vary, and most ophthalmologists in this survey did not typically refer patients to neurology or mental health services. Consequently, these patients may not be seen by established functional neurological disorder care pathways and specialist services.

Although the majority of ophthalmologists surveyed felt it appropriate to be involved in the investigation of functional visual symptoms and were confident in making a diagnosis, there was a varied response regarding confidence in discussing the psychosocial aetiology and treatment with patients. Our finding that a third of ophthalmologists would continue to see patients with functional visual symptoms, if given a choice, may reflect issues raised in comments regarding a lack of onward referral pathways and unmet training needs in discussing and managing functional visual symptoms. Ophthalmologists also highlighted the lack of time available in the clinic and appropriate space to provide a more holistic approach for these patients, which could be considered in clinical service development for functional visual symptoms in the future.

Attitudes regarding whether symptoms were “real” suggest an ongoing degree of conflation between functional visual symptoms and symptom elaboration behaviours noted in some respondents. This highlights the importance of training and increasing understanding of the aetiology of functional visual symptoms. Successful conversations with patients about the diagnosis of functional disorders, combined with providing accurate information and further sources of support, are crucial first therapeutic steps in effective management [14]. There is potential for further collaboration between ophthalmology with specialist functional disorder services to develop resources for patients and training for clinicians specific to functional visual symptoms.

We also believe that the differing responses regarding driving ability and whether patients with functional visual symptoms can be registered as sight impaired highlights the need for clear ophthalmology guidelines on these matters.

Our exploratory survey had some expected limitations of a preliminary survey. We chose a route of survey distribution that maximised dissemination to ophthalmologists of varying grades and experience. However, this meant that we could not calculate a specific response rate as we did not know how many ophthalmologists ultimately received the survey and did not respond. Though 119 respondents are not an insubstantial sample of ophthalmologists nationally, those who did respond may hold a bias of firmly held views and opinions.

We did not offer a definition of functional visual symptoms to respondents, and there may have been some doubt about which patients it included. This might have resulted in doubt about which patients to consider when responding to the survey. However, we do not consider this to be a significant criticism as this was not highlighted in free text comments and the majority of respondents were confident that they could diagnose this condition.

Ophthalmologists reported patients most commonly presented with visual loss. However, there is a broad spectrum of presentation of functional visual symptoms [12], including positive visual symptoms such as photopsia, that the survey did not capture, which require different clinical approaches. Other symptoms reported by respondents such as photophobia, diplopia or visual snow which overlaps with neurological disorders underline the role of cross-specialty collaboration in accurate diagnosis of functional disorders. This highlights the need for further clarity and consensus guidelines on the positive physical examination signs that elicit diagnosis of different functional visual symptom presentations.

This survey examined ophthalmologists’ self-reported communication and management of functional visual symptoms, which may be considerably different to how patients experience or perceive the communication regarding the condition.

This survey was limited to the UK and cannot be generalised to other countries without qualification. However, given that there are no comparable surveys of ophthalmologists in other countries, we believe this could be the basis of further, more focused surveys in other regions.

This is the first survey of UK ophthalmologists’ experience, knowledge, management and attitudes towards functional visual symptoms and has highlighted the need to establish formal links between ophthalmology (and allied services such as optometry) and services with specialist experience in the treatment of functional disorders. A prominent theme of free text comments was the lack of established pathways for psychological support for these services. Closer collaboration between neurology, psychiatry and psychological services with ophthalmology could lead to shared expertise and integrated multidisciplinary care to improve outcomes in this patient group. Though there has been a revolution in improving access to psychological therapy (IAPT) services [18] in the UK, it is unclear whether clinicians in ophthalmology services have formal links with local IAPT services or are aware of referral processes. The survey emphasises the need for future detailed research in establishing the prevalence, long-term outcomes and most effective treatments for patients with functional visual symptoms.

## Conclusion

Functional visual symptoms are disabling and relatively frequently seen in ophthalmology clinic.

Though short survey questions cannot fully explore the presentation and treatment of functional visual symptoms, our findings suggest that knowledge, service structures and current care pathways could be improved.

Improved training and consensus guidelines may offer more consistent and effective patient care while building confidence and reducing uncertainty in ophthalmological management.

This survey has demonstrated that further research is needed to understand the risk factors, long-term outcomes and most effective treatments for these patients. In addition, it has highlighted a need to establish relationships between ophthalmology services and psychological, psychiatric and neurological services with expertise in functional disorders. Ultimately, we believe that ophthalmology services are best placed to improve outcomes and develop novel multidisciplinary interventions for this patient group.

## Supplementary Information

Below is the link to the electronic supplementary material.

Supplementary file1 (PDF 22 KB)
