# Supplementary material for: How do ophthalmologists manage functional visual symptoms? A UK survey of ophthalmologists’ experience
Source: Graefes Arch Clin Exp Ophthalmol. 2021 Oct 11;260(4):1307–13. doi: 10.1007/s00417-021-05433-4 (PMC8913440; doi:10.1007/s00417-021-05433-4)
Supplement: Supplementary file 1 — Supplementary file1 (PDF 22 KB) [file 417_2021_5433_MOESM1_ESM.pdf]

National ophthalmology survey of functional visual disorders

Q1: What is your clinical grade?

- ◯ Opthalmology specialist trainee (ST1 - 3)
- ◯ Opthalmology specialist trainee (ST4 and above)
- ◯ Specialty doctor / Staff grade
- ◯ Fellow
- ◯ Associate specialist
- ◯ Consultant

Q2: What is your subspeciality?

- ☐ Not applicable
- ☐ Neuro-ophthalmology
- ☐ Paediatrics
- ☐ Medical Retina / Uveitis
- ☐ Vitreoretina
- ☐ Glaucoma
- ☐ Anterior segment
- ☐ Oculoplastics
- ☐ Primary Care Ophthalmology
- ☐ Oncology
- ☐ General

Q3: How many years have you worked in ophthalmology?

- ◯ Less than 5 years
- ◯ 5 to 10 years
- ◯ 10 to 15 years
- ◯ 15 to 20 years
- ◯ Over 20 years

Q4: Where in the UK are you currently based?

- ◯ England
- ◯ Wales
- ◯ Scotland
- ◯ Northern Ireland

Q5: Which country did you complete medical training?

- ◯ United Kingdom
- ◯ Afghanistan
- ◯ Albania
- ◯ Algeria
- ◯ Andorra
- ◯ Angola
- ◯ Anguilla
- ◯ Antigua & Barbuda
- ◯ Argentina
- ◯ Armenia
- ◯ Australia
- ◯ Austria
- ◯ Azerbaijan
- ◯ Bahamas
- ◯ Bahrain
- ◯ Bangladesh
- ◯ Barbados
- ◯ Belarus
- ◯ Belgium
- ◯ Belize
- ◯ Benin
- ◯ Bermuda
- ◯ Bhutan
- ◯ Bolivia
- ◯ Bosnia & Herzegovina
- ◯ Botswana
- ◯ Brazil
- ◯ Brunei Darussalam
- ◯ Bulgaria
- ◯ Burkina Faso
- ◯ Burundi
- ◯ Cambodia
- ◯ Cameroon
- ◯ Canada
- ◯ Cape Verde
- ◯ Cayman Islands
- ◯ Central African Republic
- ◯ Chad

- Chile
- China - Hong Kong / Macau
- Comoros
- Congo, Democratic Republic of (DRC)
- Croatia
- Cyprus
- Denmark
- Dominica
- Ecuador
- El Salvador
- Eritrea
- Eswatini
- Fiji
- France
- Gabon
- Georgia
- Ghana
- Grenada
- Guatemala
- Guinea-Bissau
- Haiti
- Hungary
- India
- Iran
- Israel and the Occupied Territories
- Ivory Coast (Cote d'Ivoire)
- Japan
- Kazakhstan
- Korea, Democratic Republic of (North Korea)
- Kosovo
- Kyrgyz Republic (Kyrgyzstan)
- Latvia
- Lesotho
- Libya
- Lithuania
- Madagascar
- Malaysia
- Mali
- Martinique
- Mauritius
- Mexico
- Monaco
- Montenegro
- Morocco
- Myanmar/Burma
- Nepal
- New Zealand
- Niger
- China
- Colombia
- Congo
- Costa Rica
- Cuba
- Czech Republic
- Djibouti
- Dominican Republic
- Egypt
- Equatorial Guinea
- Estonia
- Ethiopia
- Finland
- French Guiana
- Gambia, Republic of The
- Germany
- Greece
- Guadeloupe
- Guinea
- Guyana
- Honduras
- Iceland
- Indonesia
- Iraq
- Italy
- Jamaica
- Jordan
- Kenya
- Korea, Republic of (South Korea)
- Kuwait
- Laos
- Lebanon
- Liberia
- Liechtenstein
- Luxembourg
- Malawi
- Maldives
- Malta
- Mauritania
- Mayotte
- Moldova, Republic of
- Mongolia
- Montserrat
- Mozambique
- Namibia
- Netherlands
- Nicaragua
- Nigeria

- ◯ North Macedonia, Republic of
- ◯ Norway
- ◯ Oman
- ◯ Pacific Islands
- ◯ Pakistan
- ◯ Panama
- ◯ Papua New Guinea
- ◯ Paraguay
- ◯ Peru
- ◯ Philippines
- ◯ Poland
- ◯ Portugal
- ◯ Puerto Rico
- ◯ Qatar
- ◯ Reunion
- ◯ Romania
- ◯ Russian Federation
- ◯ Rwanda
- ◯ Saint Kitts and Nevis
- ◯ Saint Lucia
- ◯ Saint Vincent and the Grenadines
- ◯ Samoa
- ◯ Sao Tome and Principe
- ◯ Saudi Arabia
- ◯ Senegal
- ◯ Serbia
- ◯ Seychelles
- ◯ Sierra Leone
- ◯ Singapore
- ◯ Slovak Republic (Slovakia)
- ◯ Slovenia
- ◯ Solomon Islands
- ◯ Somalia
- ◯ South Africa
- ◯ South Sudan
- ◯ Spain
- ◯ Sri Lanka
- ◯ Sudan
- ◯ Suriname
- ◯ Sweden
- ◯ Switzerland
- ◯ Syria
- ◯ Tajikistan
- ◯ Tanzania
- ◯ Thailand
- ◯ Timor Leste
- ◯ Togo
- ◯ Trinidad & Tobago
- ◯ Tunisia
- ◯ Turkey
- ◯ Turkmenistan
- ◯ Turks & Caicos Islands
- ◯ Uganda
- ◯ Ukraine
- ◯ United Arab Emirates
- ◯ United States of America (USA)
- ◯ Uruguay
- ◯ Uzbekistan
- ◯ Venezuela
- ◯ Vietnam
- ◯ Virgin Islands
- ◯ Yemen
- ◯ Zambia
- ◯ Zimbabwe

Q6: Approximately what percentage (%) of new referrals do you see with functional visual symptoms?

[ ]

Q7: Approximately what percentage (%) of patients with functional visual symptoms have bilateral symptoms?

[ ]

Q8: What is the most common type of functional visual symptom you see?

- ◯ Reduced vision
- ◯ Combined reduced vision and visual field loss
- ◯ Visual Persistence Phenomena
- ◯ Photophobia
- ◯ Visual field loss
- ◯ Visual snow / "static"
- ◯ Monocular diplopia

Q9: To what degree are patients with functional visual symptoms usually impaired by their symptoms?

- ◯ No impairment
- ◯ Mild impairment
- ◯ Moderate impairment
- ◯ Severe impairment
- ◯ Extreme impairment

Q10: On average, how many appointments will a patient with functional visual symptoms have?

[ ]

Q11: What investigations do you usually perform for patients with functional visual symptoms? (more than one selection allowed)

- ☐ Humphrey Visual Field Test
- ☐ Neuro-imaging
- ☐ Optical coherence tomography (OCT)
- ☐ Other
- ☐ Goldmann Visual Field Test
- ☐ Electrodiagnostics
- ☐ Fundus Fluorescein Angiogram (FFA)

If you have chosen "other", please specify:

[ ]

Q12: Approximately what percentage (%) of ADULTS with functional visual symptoms will you follow up after diagnosis?

[ ]

Q13: Approximately what percentage (%) of CHILDREN with functional visual symptoms will you follow up after diagnosis?

[ ]

Q14: Approximately what percentage (%) of patients with functional visual symptoms get better?

[ ]

Q15: I have a good knowledge of functional visual disorders (underlying mechanisms, treatment strategies etc.)

- ◯ Strongly disagree
- ◯ Disagree
- ◯ Neither agree nor disagree
- ◯ Agree
- ◯ Strongly Agree

Q16: I received adequate education about functional visual symptoms as part of my training.

- ◯ Strongly disagree
- ◯ Disagree
- ◯ Neither agree nor disagree
- ◯ Agree
- ◯ Strongly Agree

Q17: Generally I am confident diagnosing functional visual disorders.

- ◯ Strongly disagree
- ◯ Disagree
- ◯ Neither agree nor disagree
- ◯ Agree
- ◯ Strongly Agree

Q18: These patients' symptoms are real.

- ◯ Strongly disagree
- ◯ Disagree
- ◯ Neither agree nor disagree
- ◯ Agree
- ◯ Strongly Agree

Q19: It is appropriate for me to be involved in the diagnosis of functional visual symptoms.

- ◯ Strongly disagree
- ◯ Disagree
- ◯ Neither agree nor disagree
- ◯ Agree
- ◯ Strongly Agree

Q20: It is appropriate for me to be involved in the treatment of patients with functional visual symptoms

- ◯ Strongly disagree
- ◯ Disagree
- ◯ Neither agree nor disagree
- ◯ Agree
- ◯ Strongly Agree

Q21: If I had a choice I would rather not see patients with functional visual symptoms.

- ◯ Strongly disagree
- ◯ Disagree
- ◯ Neither agree nor disagree
- ◯ Agree
- ◯ Strongly Agree

Q22: Generally I am comfortable explaining the diagnosis of a functional disorder to a patient

- ◯ Strongly disagree
- ◯ Disagree
- ◯ Neither agree nor disagree
- ◯ Agree
- ◯ Strongly Agree

Q23: I often struggle with the discussion of associated psychiatric/psychological problems.

- ◯ Strongly disagree
- ◯ Disagree
- ◯ Neither agree nor disagree
- ◯ Agree
- ◯ Strongly Agree

Q24: I am confident discussing the possibility of a functional visual disorder with a patient.

- ◯ Strongly disagree
- ◯ Disagree
- ◯ Neither agree nor disagree
- ◯ Agree
- ◯ Strongly Agree

Q25: Patients with functional visual impairment should be allowed to drive.

- ◯ Strongly disagree
- ◯ Disagree
- ◯ Neither agree nor disagree
- ◯ Agree
- ◯ Strongly Agree

Q26: Disability benefits should not be awarded to these patients because it will prevent them from getting better.

- ◯ Strongly disagree
- ◯ Disagree
- ◯ Neither agree nor disagree
- ◯ Agree
- ◯ Strongly Agree

Q27: Patients with functional visual loss can be registered as sight impaired.

- ◯ Strongly disagree
- ◯ Disagree
- ◯ Neither agree nor disagree
- ◯ Agree
- ◯ Strongly Agree

Q28: Do the majority of patients you see with functional visual symptoms tend to have other functional symptoms such as sensory loss or motor symptoms?

- ◯ Yes
- ◯ No
- ◯ Don't know

Q29: What management plan do you typically make for patients with functional visual symptoms?

- ◯ Discharge to GP (no follow up).
- ◯ Follow up in clinic.
- ◯ Refer to psychological/psychiatric services.
- ◯ Refer to neurology.

Q30: Which model do you use to explain functional visual symptoms, if patients ask? (more than one selection allowed)

- ☐ Effects of stress on the nervous system.
- ☐ Disorder of brain function.
- ☐ I can't explain it.
- ☐ Subconscious behaviour.
- ☐ Mind over matter.
- ☐ Other.

Q31: Please enter any comments on the survey, or issues you would like to raise with regard to functional visual symptoms in ophthalmology.
